# RIG-I: a multifunctional protein beyond a pattern recognition receptor

**DOI:** 10.1007/s13238-017-0431-5

**Published:** 2017-06-08

**Authors:** Xiao-xiao Xu, Han Wan, Li Nie, Tong Shao, Li-xin Xiang, Jian-zhong Shao

**Affiliations:** 1 Key Laboratory for Cell and Gene Engineering of Zhejiang Province, College of Life Sciences, Zhejiang University, Hangzhou, 310058 China; 2 Laboratory for Marine Biology and Biotechnology, Qingdao National Laboratory for Marine Science and Technology, Qingdao, 266235 China

**Keywords:** RIG-I, viral RNA, endogenous RNA, immunity, cancer

## Abstract

It was widely known that retinoic acid inducible gene I (RIG-I) functions as a cytosolic pattern recognition receptor that initiates innate antiviral immunity by detecting exogenous viral RNAs. However, recent studies showed that RIG-I participates in other various cellular activities by sensing endogenous RNAs under different circumstances. For example, RIG-I facilitates the therapy resistance and expansion of breast cancer cells and promotes T cell-independent B cell activation through interferon signaling activation by recognizing non-coding RNAs and endogenous retroviruses in certain situations. While in hepatocellular carcinoma and acute myeloid leukemia, RIG-I acts as a tumor suppressor through either augmenting STAT1 activation by competitively binding STAT1 against its negative regulator SHP1 or inhibiting AKT-mTOR signaling pathway by directly interacting with Src respectively. These new findings suggest that RIG-I plays more diverse roles in various cellular life activities, such as cell proliferation and differentiation, than previously known. Taken together, the function of RIG-I exceeds far beyond that of a pattern recognition receptor.

## INTRODUCTION

RIG-I is a highly important cytosolic pattern recognition receptor (PRR) involved in sensing RNA virus infection and inducing interferon (IFN) production. This gene was first identified in the all-trans-retinoic acid-induced differentiation of acute promyelocytic leukemia (APL) cells. In the past decades, the structure, antiviral signaling pathway, and function of RIG-I in innate antiviral immunity were well documented (Yoneyama et al., 2004; Seth et al., 2005; Yoneyama et al., 2005; Saito et al., 2007; Kowalinski et al., 2011). However, the long-held paradigm of RIG-I serving as a PRR has been extended because of emerging new findings on the biological functions of RIG-I in other cellular activities (Zhang et al., 2008; Jiang et al., 2011; Boelens et al., 2014; Hou et al., 2014; Zeng et al., 2014). For example, besides recognizing exogenous viral RNAs, RIG-I closely associates with various endogenous RNAs, such as microRNAs, snRNAs, and endogenous retroviruses (ERVs) in certain situations, which participates in many important cellular activities, such as the therapy resistance and expansion of cancer cells, and the activation of T cell-independent B cells (Karlsen et al., 2013; Boelens et al., 2014; Zeng et al., 2014; Zhao et al., 2015; Ranoa et al., 2016). RIG-I also acts as a tumor suppressor in acute myeloid leukemia (AML) and hepatocellular carcinoma (HCC) (Jiang et al., 2011; Hou et al., 2014; Li et al., 2014). These observations suggest that RIG-I is a multifunctional protein that functions far beyond being a PRR, and the biological functions of RIG-I are more complicated than previously believed. This review depicts these new findings for a deepened understanding of RIG-I.

## WIDER RECOGNITION IN ANTIVIRAL IMMUNITY

 RIG-I has been previously identified to recognize relatively short duplexed regions of RNAs with blunt-ended 5′-triphosphate or 5′-diphosphate, which are often present at the end of genomic RNAs of (+) ssRNA viruses (Kato et al., 2005; Chang et al., 2006; Kato et al., 2006; Le Goffic et al., 2007; Loo et al., 2008). Some (−) ssRNA viruses that do not form dsRNA during infection can still be detected by RIG-I (Yoneyama et al., 2005; Kato et al., 2006; Pichlmair et al., 2006). This detection was rendered possible by the highly complementary 5′- and 3′-sequences that viral ssRNAs contain because these highly complementary sequences could form short double-stranded structures with perfectly blunt ends. Recently, an A/U-rich motif in the 3′-untranslated region of the hepatitis C virus genome was found necessary for the effective detection by RIG-I (Saito et al., 2008). Furthermore, the purified HIV-1 RNA can also be recognized by RIG-I. The viral RNA with uncapped 5′-triphosphate end at the early stage after viral infection preferentially associates with RIG-I. Meanwhile, the monomeric RNA existing in defective HIV-1 particles induces a stronger IFN expression than that of dimeric RNA. Several stem-loop structures containing A/U-rich sequences in the 5′-untranslated region of the HIV genome were found to involve in RIG-I activation (Solis et al., 2011). Besides, many other RNAs, such as poly-I:C, poly-U/UC RNA, 2′,5′-polyadenylic acid formed by RNase L digestion, and 5′pppAU-RNA transcribed by RNA polymerase III, are associated with RIG-I (Yoneyama et al., 2004; Hornung et al., 2006; Malathi et al., 2007). The plasticity of RIG-I in RNA recognition provides clues for uncovering additional potential ligands. Intriguingly, some viral variants can escape from host defense by avoiding being recognized by RIG-I. For example, in an influenza A virus (IAV) variant, Glu residue replacement in the polymerase subunit PB2 with Lys increases the affinity of the mutant subunit (PB2-627K) to the IAV nucleocapsid. This enhancement then prevents the RIG-I from accessing the viral RNA and thereby limits antiviral restriction (Weber et al., 2015). RIG-I also directly inhibits the replication of the hepatitis B virus (HBV) by competitively recruiting pregenomic RNA (pgRNA) from the HBV polymerase. In this process, RIG-I binds to the 50-ε stem-loop region of the pgRNA and then counteracts the interaction of the HBV polymerase with the 50-ε region. This occurrence consequently inhibits viral replication. The association of RIG-I with pgRNA also leads to the induction of type III IFN, which plays a negative role in viral proliferation (Sato et al., 2015). Moreover, the increased recognition coverage of RIG-I may increase the receptor’s diverse functions in antiviral immunity.

## INTERACTION WITH ENDOGENOUS RNAS IN DIFFERENT SITUATIONS

**Figure 1:**
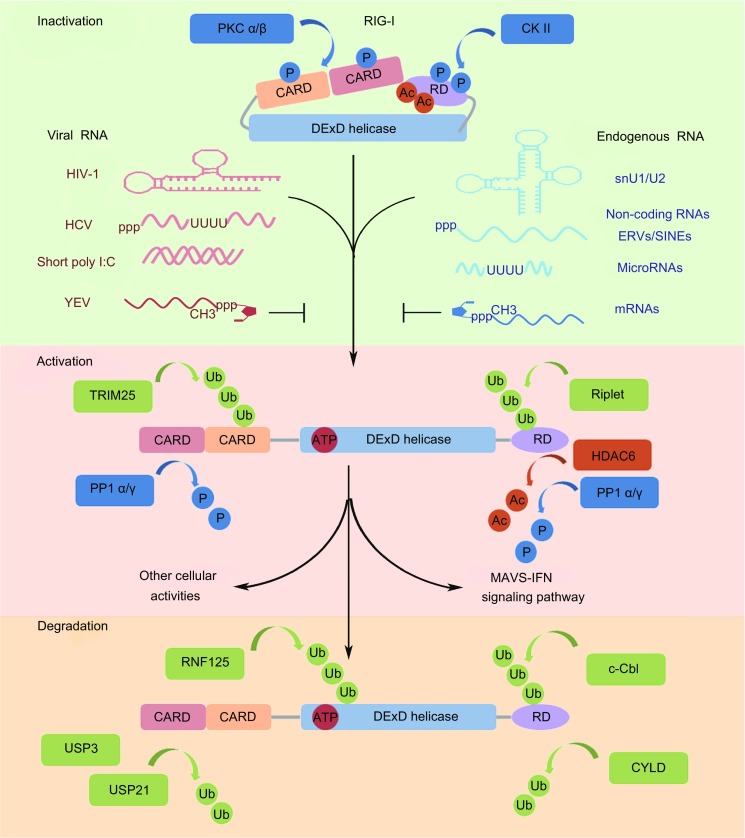
**Various RNA ligands and post-translational regulations of RIG-I**. Given the deepened and broadened study of RIG-I, various RNAs have been found to be recognized by RIG-I, including classical viral RNAs and some endogenous RNAs. The mRNAs are equipped with N1-2′O-methylation of the ^m7^G cap structure to block RIG-I activation, which is also utilized by the yellow fever virus. Besides, RIG-I activation is accurately orchestrated by the post-translational modifications, such as polyubiquitination, phosphorylation, and deacetylation

Besides viral RNAs, endogenous RNAs, including various non-coding RNAs and ERVs, may be recognized by RIG-I (Fig. 1). For instance, the RIG-I in breast cancer (BrCa) cells is activated by the exosomes released from stromal cells. Nearly 75% of RNAs contained in the exosomes have been determined to be non-coding RNAs. Most of these RNAs are transposable or repetitive elements originating from the viral genome. These RNAs participate in the RIG-I-mediated IFN signaling pathway, which facilitates the therapy resistance and expansion of BrCa cells (Boelens et al., 2014). Furthermore, snRNA U1 and U2, which are the two key components of spliceosomes, can interact with RIG-I in human colorectal carcinoma (HCT116) cells. The snRNA U1 and U2 are normally located in the nucleus during RNA splicing. Once HCT116 cells are exposed to ionizing radiation (IR), snRNA U1 and U2 will be translocated into the cytoplasm, where they bind RIG-I to activate the IFN pathway. This process then enhances the IR-induced cytotoxic responses (Ranoa et al., 2016). Likewise, some microRNAs, such as miR-136 and miR-145, were closely associated with RIG-I. MiR-136 was significantly upregulated in H5N1- and VSV-infected human lung epithelial cells. Elevated miR-136 then interacts with RIG-I to induce the expression of IFN-β and IL-6, both of which inhibit viral replication (Zhao et al., 2015). The interaction of miR-145 with RIG-I triggers the off-target immune responses of mesenchymal stem cells by upregulating IFN stimulatory genes (ISGs) following the delivery of miR-145 by liposome (Karlsen et al., 2013). Strikingly, endogenous retroelements, including ERVs and short interspersed elements (SINEs), can activate RIG-I. In mouse models, RIG-I promoted T cell-independent B cell activation by engaging BCR with TI-2 antigens to induce ERV transcription through NF-κB activation. ERV transcripts then activate RIG-I and cGAS in the cytoplasm. A positive feedback is ultimately released to facilitate IgM production (Zeng et al., 2014). Besides, SINEs could also function as endogenous ligands for RIG-I when the SINEs are transcribed by polymerase III under cellular and environmental stresses (Mu et al., 2016).

## SELECTIVE RECOGNITION FOR CELLULAR RNAS

Except for the endogenous RNAs recognized by RIG-I, there are abundant other cellular RNAs, such as mRNAs and rRNAs in the cytoplasm, which must utilize some unique strategies to avoid themselves being recognized by RIG-I. Actually, the eukaryotic mRNAs are equipped with the ^m7^G cap structure, which is essential in avoiding RIG-I recognition (Schuberth-Wagner et al., 2015). Three forms of cap structures (namely, caps 0, 1, and 2) exist in eukaryotic mRNA. The major portion of the eukaryotic mRNA cap structure corresponds to cap 1. Cap 1 consists of an N7-methyl guanosine and a 2′O-methyl group at the first nucleotide (N1) position. N1-2′O-methylation is crucial for preventing RIG-I from being activated by self-mRNAs because the knockdown of N1-2′O-methylteansferase induces a strong RIG-I activation in the absence of exogenous stimuli. Furthermore, a conserved H830 residue is important for the tolerance of RIG-I to mRNAs. Replacing H830 of RIG-I by alanine leads to the indistinguishable binding of mRNAs with or without N1-2′O-methylation. This occurrence causes RIG-I to lose the ability to discriminate “non-self” from “self.” Intriguingly, yellow fever virus encodes a viral 2′O-methyltransferase to achieve the N1-2′O-methylation of its genomic RNA. The genomic RNA then mimics the structure of endogenous mRNA to escape recognition by RIG-I. Furthermore, the unintentional recognition of self-RNAs can also be impeded by the ATP hydrolysis of RIG-I (Lassig et al., 2015). Binding to ATP through K270 of the superfamily II (SF2) domain is important for RIG-I activation. By contrast, a defect in ATP breakdown may result in constitutive signal transduction and autoimmune diseases. In a study on the Singleton-Merten syndrome, an ATP-hydrolysis-deficient RIG-I (E373Q) was retained in an ATP-bound state, which spontaneously senses enriched self-RNAs. This ATP-hydrolysis-deficient RIG-I (E373Q) is an amino-acid-substitution mutant at site 373 with slowed-down ATP hydrolysis. This E373Q mutant subsequently combines with additional endogenous RNAs, particularly the dsRNA expansion segments of the ribosomal large subunit, such as 28S rRNA. A contiguous stretch of seven G:C/C:G base pairs in 28S rRNA was identified to be essential in the recognition by ATPase-deficient RIG-I. In the presence of ATP, RIG-I preferentially binds RNAs lacking base-paired ends. Overall, ATP hydrolysis is not only essential for RIG-I activation, but also for self-RNA immune tolerance.

## POST-TRANSLATIONAL REGULATION OF RIG-I ACTIVATION

RIG-I activation is tightly regulated by post-translational modifications, such as polyubiquitination and phosphorylation. The polyubiquitination of RIG-I includes K-63- or K-48-linked polyubiquitination at different sites, which controls the “turn on” or “turn off” of RIG-I activation, respectively. TRIM25, MEX3C, and Riplet catalyze the K-63-linked polyubiquitination of RIG-I in CARD and RD domains at multiple lysine residues. This polyubiquitination is essential for recruiting RIG-I to MAVS with the help of a mitochondrion-targeting chaperone 14-3-3ε (Gack et al., 2007; Oshiumi et al., 2013; Kuniyoshi et al., 2014). RNF125 and c-Cbl recruited by Siglec-G with the help of SHP2 catalyze the K-48-linked polyubiquitination at Lys-362, Lys-461, and Lys-813 residues. This modification leads to the proteasomal degradation of RIG-I and attenuates antiviral signals (Arimoto et al., 2007; Chen et al., 2013). However, a recent study revealed that E3 ubiquitin ligase FBXW7 stabilized RIG-I by interacting with SHP2 to cause the latter’s ubiquitination and degradation. As a result, the SHP2/c-Cbl complex, which mediates RIG-I degradation, was disrupted (Song et al., 2017). Besides, apart from the ubiquitin chains that covalently conjugate with RIG-I, the unanchored K63-ubiquitin chains, which do not conjugate to any target protein, can also promote RIG-I activation (Zeng et al., 2010). Several deubiquitination enzymes, such as CYLD, USP3, and USP21, which remove the K-63-linked polyubiquitination from RIG-I, serve as negative feedback regulators for RIG-I activation (Friedman et al., 2008; Cui et al., 2014; Fan et al., 2014). RIG-I undergoes phosphorylation at Ser-8 and Thr-170 residues by PKC-α and PKC-β at normal conditions. These modifications hinder the TRIM25 binding to the first CARD and the K-63-linked polyubiquitination of the Lys-172 of RIG-I. Ultimately, the RIG-I signaling pathway is inhibited (Maharaj et al., 2012). Moreover, the Thr-770 and Ser-854–855 located at the RD domain can be phosphorylated by casein kinase II (CK II) to enable the autoinhibition of RIG-I (Sun et al., 2011). Upon virus infection, the interaction between RD and CARD is disassociated by the PP1α- and PP1γ-mediated dephosphorylations of RIG-I. This dephosphorylation simultaneously allows the TRIM25 binding and ubiquitination process and ultimately helps in initiating the antiviral signaling pathway (Wies et al., 2013). Importantly, recent studies also revealed that deacetylation is equally crucial to the activation of RIG-I and induction of downstream signaling. The acetylated Lys-909 at the CTD domain of RIG-I may form a hydrogen bond with Lys-907. This bond formation prevents the interaction of Lys-907 with the phosphate of 5′ppp dsRNA. Upon RNA virus infection, the HDAC6-mediated deacetylation of Lys-909 will release Lys-907 for phosphate binding and promote the RIG-I sensing of viral RNAs (Choi et al., 2016). In addition, the deacetylation of Lys-909 and Lys-858 by HDAC6 also facilitates the oligomerization of RIG-I, which subsequently forms a nucleus onto which the MAVS can aggregate and polymerize to initiate the downstream signaling pathway (Liu et al., 2016a). Except for the regulators mentioned in the above modifications of RIG-I, some other regulatory proteins are also involved in RIG-I activation by direct protein interaction. For example, the PARP-13 shorter isoform (ZAPS) induced by 5′-triphosphate RNA serves as a positive feedback regulator that promotes the oligomerization and ATPase activity of RIG-I possibly by stabilizing the RNA–RIG-I complex. Consequently, the downstream signaling pathway is enhanced to eliminate viral infection (Hayakawa et al., 2011). Certainly, other regulatory mechanisms, including post-transcriptional modifications, microRNAs, and crosstalk with NOD2 and autophagy signaling pathways, also operate together to orchestrate the accurate actions of RIG-I in response to diversified stimulations.

## MULTIPLE ROLES IN CELLULAR DEVELOPMENT AND CANCERS

**Figure 2:**
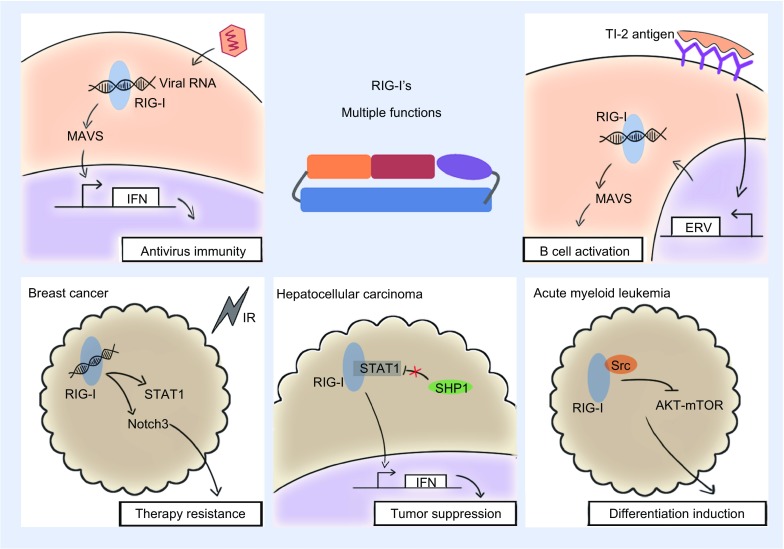
**Multiple functions of RIG-I**. RIG-I equally exerts versatile functions in immunity and cancer along with its sensing ability of diverse RNAs. For example, RIG-I can initiate IFN synthesis in antiviral responses and promote IgM production in TI-2 B cell activation. RIG-I can also simultaneously help cells resist therapy and facilitate cell expansion in BrCa, suppress tumor growth in HCC and induce differentiation in AML

RIG-I was initially identified to play a regulatory role in the differentiation of granulocytes from APL cells induced by all-trans-retinoic acid. Subsequently, RIG-I was found to be an essential negative regulator of myeloid development, in which the RIG-I expression was developmentally up-regulated along with myelopoiesis (Zhang et al., 2008). RIG-I gene defect in mice may disrupt physiological myelopoiesis, especially granulopoiesis, resulting in a progressive myeloproliferative disorder. Investigation of the underlying mechanism showed that RIG-I regulates the proliferation and survival of granulocytes by down-regulating the expression level of IFN consensus sequence binding protein (ICSBP), a major transcription factor regulating myeloid cell differentiation. In view of the above observations, RIG-I was recently identified as a tumor suppressor in the study of the terminal granulocytic differentiation of AML (Fig. 2). In this disease model, the defect of RIG-I leads to the progression of AML along with the evident decline in the expression level of ICSBP. In addition, RIG-I also enhances the expression of other numerous IFN stimulatory genes (ISGs) by promoting STAT1 activation in a MAVS-independent manner. Thus, the efficacy of IFN-/RA-induced differentiation and proliferation restriction of leukemia cells is amplified (Jiang et al., 2011). Moreover, another mechanism was found, in which RIG-I inhibited AKT-mTOR signaling pathway by directly interacting with the Src protein through the PxxP motif. This result restricts the proliferation of myeloid progenitors and the *in vivo* repopulating capacity of leukemia cells (Li et al., 2014). Simultaneously, a similar function of RIG-I was detected in HCC, wherein RIG-I promotes STAT1 activation by competing the SH2-TA binding domain with SHP1. SHP1 is a negative regulator of STAT1 that can catalyze the dephosphorylation of STAT1 after binding with an identical domain. The enhanced activation of STAT1 then induces the expression of downstream ISGs, especially those associated with apoptosis, such as TRAIL, PML, XAF1, and OAS1. These ISGs then control HCC carcinogenesis and progression (Hou et al., 2014). Interestingly, this mechanism is subjected to full use by miR-545 that targets RIG-I and down-regulates its expression to activate PI3K/Akt signaling and promote HCC development (Liu et al., 2016c). However, an opposite effect was achieved in pancreatic ductal adenocarcinoma (PDAC) by the miR-545-mediated down-regulation of RIG-I. In this case, the low miR-545 expression level and the high RIG-I protein level in PDAC tissues promote tumor cell growth and are both correlated with low survival rate, the mechanism of which remains to be explored (Song et al., 2014). In contrast to the antitumor effect, RIG-I serves a facilitative function in BrCa therapy resistance and BrCa cell expansion (Boelens et al., 2014). In BrCa cells, RIG-I triggers STAT1-dependent signaling pathway by the stimulation of endogenous RNAs in the exosomes released by stromal cells. Meanwhile, stromal cells also induce NOTCH3 activation in BrCa cells, which exert transcriptional functions to stimulate NOTCH target genes that are simultaneously facilitated by the activated STAT1.

Notably, the anti-tumor ability of RIG-I has been widely used in the immune therapy of other cancers. For example, in HCT116 cells, RIG-I can be activated by snRNA U1/2, which are translocated from the nucleus into the cytoplasm in cells stimulated by IR. This occurrence ultimately leads to tumor growth arrest and cell death (Ranoa et al., 2016). In prostate cancer cells, the RIG-I-MAVS signaling pathway can be activated by transfecting with inactivated Sendai virus to induce the secretion of IFNs and induction of several ISGs, which result in cancer cell-selective apoptosis (Kawaguchi et al., 2009; Kato et al., 2010; Matsushima-Miyagi et al., 2012; Liu et al., 2016b). Furthermore, synthetic analog of dsRNA can be utilized to trigger the RIG-I-dependent anticancer immunity in prostate cancer (Palchetti et al., 2015). Similar therapies have also been found in pancreatic cancer, where the synthetic siRNA used for silencing the TGF-β gene can simultaneously activate RIG-I and result in enhanced antitumor efficacy against pancreatic cancer (Ellermeier et al., 2013)

## FUTURE OUTLOOK

A comprehensive view of the roles of RIG-I in recognizing endogenous RNAs and multiple functions in immunity and cancer will greatly extend the current knowledge on this gene. RIG-I has long been known to serve as a PRR for viral detection. However, this receptor was also found to serve an extensive function as an RNA-responsiveness protein for various cellular activities, including cell proliferation and development. This additional knowledge will also provide promising prospective targets for incurable diseases. Further studies are expected to focus on the accurate actions and mechanisms of RIG-I in its diversified functions, as well as the crosstalk between different biological processes. These further investigations would increase the understanding of RIG-I-mediated biology and diseases. Additional work could also help determine the overall functional and evolutionary correlation among RIG-I, ERVs, and current retroviruses.
